# Pregnancy Outcomes after a Mass Vaccination Campaign with an Oral Cholera Vaccine in Guinea: A Retrospective Cohort Study

**DOI:** 10.1371/journal.pntd.0004274

**Published:** 2015-12-29

**Authors:** Lise Grout, Isabel Martinez-Pino, Iza Ciglenecki, Sakoba Keita, Alpha Amadou Diallo, Balla Traore, Daloka Delamou, Oumar Toure, Sarala Nicholas, Barbara Rusch, Nelly Staderini, Micaela Serafini, Rebecca F. Grais, Francisco J. Luquero

**Affiliations:** 1 Epicentre, Paris, France; 2 World Health Organization, Geneva, Switzerland; 3 European Programme for Intervention Epidemiology Training (EPIET), European Centre for Disease Prevention and Control (ECDC), Stockholm, Sweden; 4 Médecins Sans Frontières, Geneva, Switzerland; 5 Ministry of Health and Public Hygiene, Guinea; 6 Médecins Sans Frontières, Conakry, Guinea; 7 Johns Hopkins Bloomberg School of Public Health, Baltimore, Maryland, United States of America; George Washington University, UNITED STATES

## Abstract

**Introduction:**

Since 2010, WHO has recommended oral cholera vaccines as an additional strategy for cholera control. During a cholera episode, pregnant women are at high risk of complications, and the risk of fetal death has been reported to be 2–36%. Due to a lack of safety data, pregnant women have been excluded from most cholera vaccination campaigns. In 2012, reactive campaigns using the bivalent killed whole-cell oral cholera vaccine (BivWC), included all people living in the targeted areas aged ≥1 year regardless of pregnancy status, were implemented in Guinea. We aimed to determine whether there was a difference in pregnancy outcomes between vaccinated and non-vaccinated pregnant women.

**Methods and Findings:**

From 11 November to 4 December 2013, we conducted a retrospective cohort study in Boffa prefecture among women who were pregnant in 2012 during or after the vaccination campaign. The primary outcome was pregnancy loss, as reported by the mother, and fetal malformations, after clinical examination. Primary exposure was the intake of the BivWC vaccine (Shanchol) during pregnancy, as determined by a vaccination card or oral history. We compared the risk of pregnancy loss between vaccinated and non-vaccinated women through binomial regression analysis. A total of 2,494 pregnancies were included in the analysis. The crude incidence of pregnancy loss was 3.7% (95%CI 2.7–4.8) for fetuses exposed to BivWC vaccine and 2.6% (0.7–4.5) for non-exposed fetuses. The incidence of malformation was 0.6% (0.1–1.0) and 1.2% (0.0–2.5) in BivWC-exposed and non-exposed fetuses, respectively. In both crude and adjusted analyses, fetal exposure to BivWC was not significantly associated with pregnancy loss (adjusted risk ratio (aRR = 1.09 [95%CI: 0.5–2.25], p = 0.818) or malformations (aRR = 0.50 [95%CI: 0.13–1.91], p = 0.314).

**Conclusions:**

In this large retrospective cohort study, we found no association between fetal exposure to BivWC and risk of pregnancy loss or malformation. Despite the weaknesses of a retrospective design, we can conclude that if a risk exists, it is very low. Additional prospective studies are warranted to add to the evidence base on OCV use during pregnancy. Pregnant women are particularly vulnerable during cholera episodes and should be included in vaccination campaigns when the risk of cholera is high, such as during outbreaks.

## Author Summary

Pregnant women are at high risk of complications and fetal deaths when ill with cholera. However, they have been excluded in most cholera vaccination campaigns because of the lack of safety data on oral cholera vaccines during pregnancy. This study aimed to determine if the risk of pregnancy loss changed after the administration of the oral cholera vaccine in Guinea in 2012. We visited all households in Boffa and Koba sub-prefectures, where the vaccination campaign took place, and enrolled a total of 2,493 women in the study. In this large retrospective cohort, we found no association between fetal exposure to the cholera vaccine and the risk of pregnancy loss or malformation. Pregnant women are particularly vulnerable during a cholera episode and should be included in vaccination campaigns when the risk of cholera is high, such as during the outbreaks.

## Introduction

Cholera represents a risk of complications for pregnant women and their fetus. Published literature reports fetal loss rates during cholera episodes of between 2% and 36% [1–7]. However, comparison of pregnancy outcomes among different reports is difficult, due to differences in inclusion criteria, treatment provided, and access to care. Although the exact cause of fetal death during a cholera episode has not yet been identified, several studies suggest an association between fetal loss and the degree of dehydration and hypovolemia [2,4–7].

In cholera-endemic countries, the World Health Organization (WHO) recommends vaccination “for groups that are especially vulnerable to severe disease and for which the vaccines are not contraindicated, such as pregnant women and HIV-infected individuals” [8]. WHO has prequalified two oral cholera vaccines (OCV), both consist of killed whole-cells of *V*. *cholerae*. One consists of several strains of *V*. *cholerae* O1 and a recombinant B subunit of the cholera toxin (WC-rBS, marketed as Dukoral); the other contains strains from both serogroups O1 and O139, but no component of the cholera toxin (BivWC, marketed as Shanchol) [8]. According to the package inserts, neither vaccine is contraindicated in pregnant women, but only recommended when the potential benefits are considered higher than the risk. Inactivated OCVs are unlikely to have a harmful effect on fetal development as the killed bacteria in the vaccine do not replicate, the vaccine antigens act locally in the gastrointestinal mucosa, are not absorbed and do not enter the maternal or fetal circulation. In addition, the vaccines do not trigger systemic reactions (e.g. fever) associated with miscarriage in early pregnancy [9].

Pre-licensure studies and post-marketing surveillance suggest that Dukoral has a good safety profile when used during pregnancy [4] and inadvertent vaccination of pregnant women with the vaccine during a mass vaccination campaign in Zanzibar in 2009 was not associated with any harmful effects [9]. However, pregnant women have been excluded systematically from most other cholera vaccination campaigns because of the weak data on safety during pregnancy for Dukoral and the absence of safety data during pregnancy for Shanchol [10]. Shanchol has several advantages compared with Dukoral for public health use. The vaccine is cheaper, has a lower storage volume and does not require water for administration. Thus, understanding the safety of BivWC during pregnancy will provide essential information for its future use throughout the cholera-endemic world.

The Ministry of Health and Public Hygiene (MHPH) of Guinea, with the support of Médecins Sans Frontières (MSF), carried out mass OCV campaigns using BivWC in 2012 in Boffa and Forécariah Prefectures as part of a comprehensive response to a cholera epidemic that was spreading in remote rural areas with limited access to health facilities [11,12]. These campaigns targeted all people aged one year and above living in the target areas [11,12]. Pregnant women were not excluded from the target population.

In order to assess whether there was a difference in pregnancy outcomes between women who exposed their fetus to OCV and those who did not, we report the results of a retrospective cohort study, which compared the incidence of pregnancy losses (miscarriages and stillbirths) and malformations between these two groups.

## Methods

**Fig 1:**
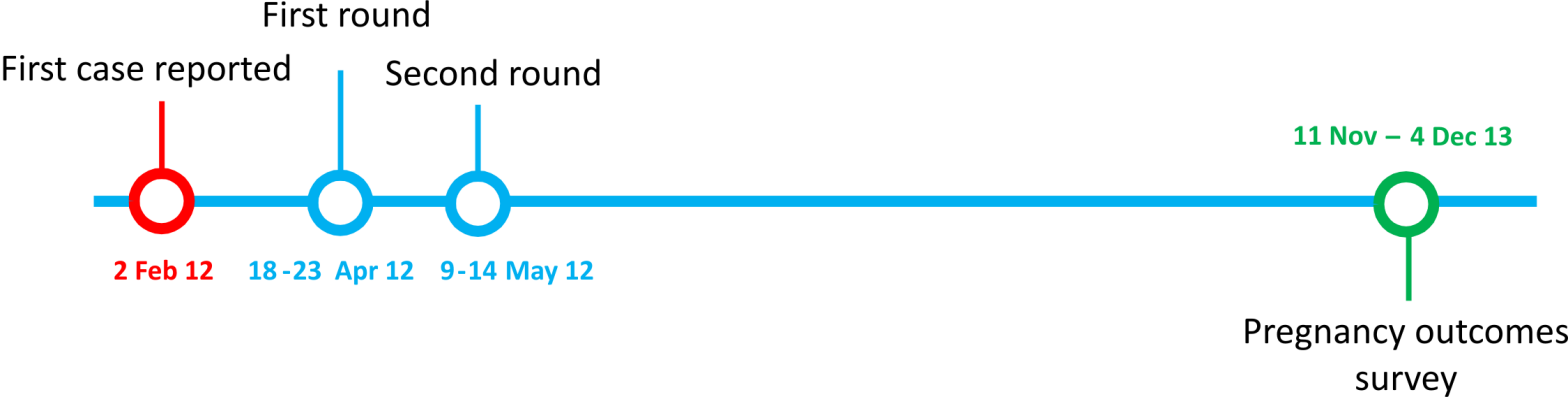
Timeline of the cholera vaccination campaign and field surveys in Boffa prefecture, Guinea, 2012 and 2013.

The study took place in Boffa Prefecture of Guinea where six sub-prefectures bordering the ocean were targeted for cholera vaccination campaigns. All residents one year of age and above were offered a first dose from 18 to 23 April and a second dose from 9 to 14 May 2012 (Fig 1). The retrospective cohort study was conducted in two of these sub-prefectures (Koba and Boffa), since the association between vaccine exposure and pregnancy outcomes was assumed independent of the sub-prefecture.

### Study population

Women were included in the study if they were residents of the Koba and Boffa subprefectures, were 15 to 49 years old, were pregnant in 2012 (i.e., conception and/or birth occurred that year) and if they (or their guardians for minor participants) provided informed consent. Exclusion criteria were non-residence in Boffa prefecture at the time of the vaccination campaign, absence from the home after two visits, lack of knowledge of their vaccination status, and refusal to participate.

### Sample size

Based on published literature [13–16], we assumed a 10% incidence of pregnancy loss, an unexposed/exposed ratio of 0.3 (based on 77% of pregnant women vaccinated in the vaccination coverage survey), an alpha error of 0.05, and a statistical power of 0.8. Thus, 1,200 vaccinated pregnant women and 360 non-vaccinated pregnant women were necessary to estimate a 1.5 increase in the risk of pregnancy loss among vaccinated women.

### Data collection

All interviewers and supervisors were recruited locally and received theoretical and practical training. They visited all households (defined as a group of individuals living under the same roof and regularly sharing the same meals). Interviewers revisited households later in the day where no one was at home. If there was no response the second time, the household was skipped. Interviewers asked the head of household for the number of women between 16 and 50 years old living in the household, and the number of women who were pregnant in 2012, irrespective of pregnancy outcome. They obtained written informed consent from the women who were pregnant in 2012 and conducted face-to-face interviews in the local language.

A standardized pre-tested questionnaire was used to collect inclusion criteria, socio-demographic data, information about the pregnancy, pregnancy history and other risk factors for pregnancy loss. Vaccination status was assessed at the end of the questionnaire. Interviewers also completed a questionnaire to determine the health condition of live-born babies. Mothers and children were referred to a pediatrician if the questionnaire elicited concerns. The pediatrician completed a clinical examination and determined if the child was ill or presented any malformation. The medical team was also in charge of patient management (i.e. ambulatory treatment or transfer to hospital), if needed.

### Data analysis

The primary outcome of the study was the incidence of pregnancy loss, defined as any loss of a product of conception after the woman recognizes she is pregnant. Secondary outcomes included the incidence of miscarriage, stillbirth and malformation in live children. A miscarriage was defined as a loss of a clinically recognized pregnancy before the end of the fifth month of gestation and a stillbirth as the delivery of a dead fetus (without pulse) after the end of the fifth month of gestation. These outcomes were reported orally by the mother and verified by documentation when possible. A malformation was defined as a physical defect in a live infant that was identified by the study pediatrician.

Primary exposure was defined as the intake of OCV during pregnancy. Participants were asked whether they had been vaccinated and, if so, to show their vaccination cards. A fetus was considered exposed if the mother was pregnant during the campaign, received at least one dose of OCV (card-confirmed or reported orally), and at least one dose was received after the estimated date of conception and before the date of birth or fetal loss. Date of birth was reported orally and verified by documentation when possible. The date of conception was calculated by subtracting the duration of the pregnancy (reported orally or confirmed by documentation) from the date of birth or fetal loss. When date of birth or fetal loss was unknown, the mother was asked if she was pregnant during the vaccination campaign.

The primary data analysis included women who were pregnant during the mass vaccination campaign. Descriptive analysis of these women was stratified by their vaccination status. Qualitative and quantitative variables were compared, respectively, through Fisher and Wilcoxon tests. The fetus was then considered as the unit of analysis since some women had multiple pregnancies. We calculated crude cumulated incidence of pregnancy loss as the number of pregnancy losses divided by the number of conceived fetuses. We compared the risk of pregnancy loss through a binomial regression. Possible confounders were variables for which p-values were less than 0.20 in the bivariate analysis. We obtained an adjusted estimate of relative risk (aRR) of pregnancy loss and its 95% confidence interval (95%IC) according to OCV exposure using a forward stepwise procedure. The interaction between trimester of the pregnancy on 18 April 2012 and primary exposure was tested. All covariates significantly associated with the risk of a pregnancy loss (p-value <0.05) or those improving model fit (based on Bayesian Information Criterion) were retained in the final model. Women with missing data were excluded from the analysis.

In a secondary analysis, the same procedure was applied to other negative outcomes (miscarriages, stillbirths and malformations). Fetuses born to mothers who had been pregnant for more than five months on 18 April 2012 were excluded from the analysis of the risk of miscarriage. Fetuses who did not complete five months of gestation were excluded from the analysis of the risk of stillbirth. Children who were not alive at the time of the survey (fetal or perinatal deaths) were excluded from the analysis of the risk of malformations.

A bias-indicator analysis of fetuses conceived in 2012 *after* the second vaccination round was conducted to assess bias from possible misclassification of the women vaccination status or fetal outcome. This analysis again compared pregnancy outcomes of woman who had been vaccinated during the campaign with women who did not receive the vaccine. Since OCV intake before conception is not supposed to have an effect on pregnancy outcome, this analysis provides information about possible information bias.

Since the exact dates of vaccination, conception and fetal lost were mainly estimates, we conducted sensitivity analyses by excluding all fetuses born or lost within seven days of the first round of the vaccination campaign and those whose estimated date of conception was within two weeks following the first round of the campaign.

Data entry was performed using EpiData 3.1 (EpiData Association, Denmark) and data analysis was performed using Stata 12.0 (College Station, USA).

### Ethical considerations

This study was conducted according to the ethical principles for research on human subjects, described in the Helsinki Declaration, and in accordance with international principles and guidelines for biomedical research involving human subjects, published by the Council for International Organizations of Medical Sciences. The study protocol was approved by National Ethics Committee of the Republic of Guinea and the Médecins Sans Frontières Ethics Committee.

Each woman (or her legal representative) received the information on the methods and potential risks and benefits of the study. The participant or her representative signed an informed consent form after being informed that participation in the study was voluntary and that she could withdraw from the study at any time. Anonymity and confidentiality of collected data were ensured throughout the study. If there was any suspected illness in the live-born babies, they were referred to the pediatrician, were treated or referred and hospitalized, if needed. All treatment was provided free of charge.

## Results

**Fig 2:**
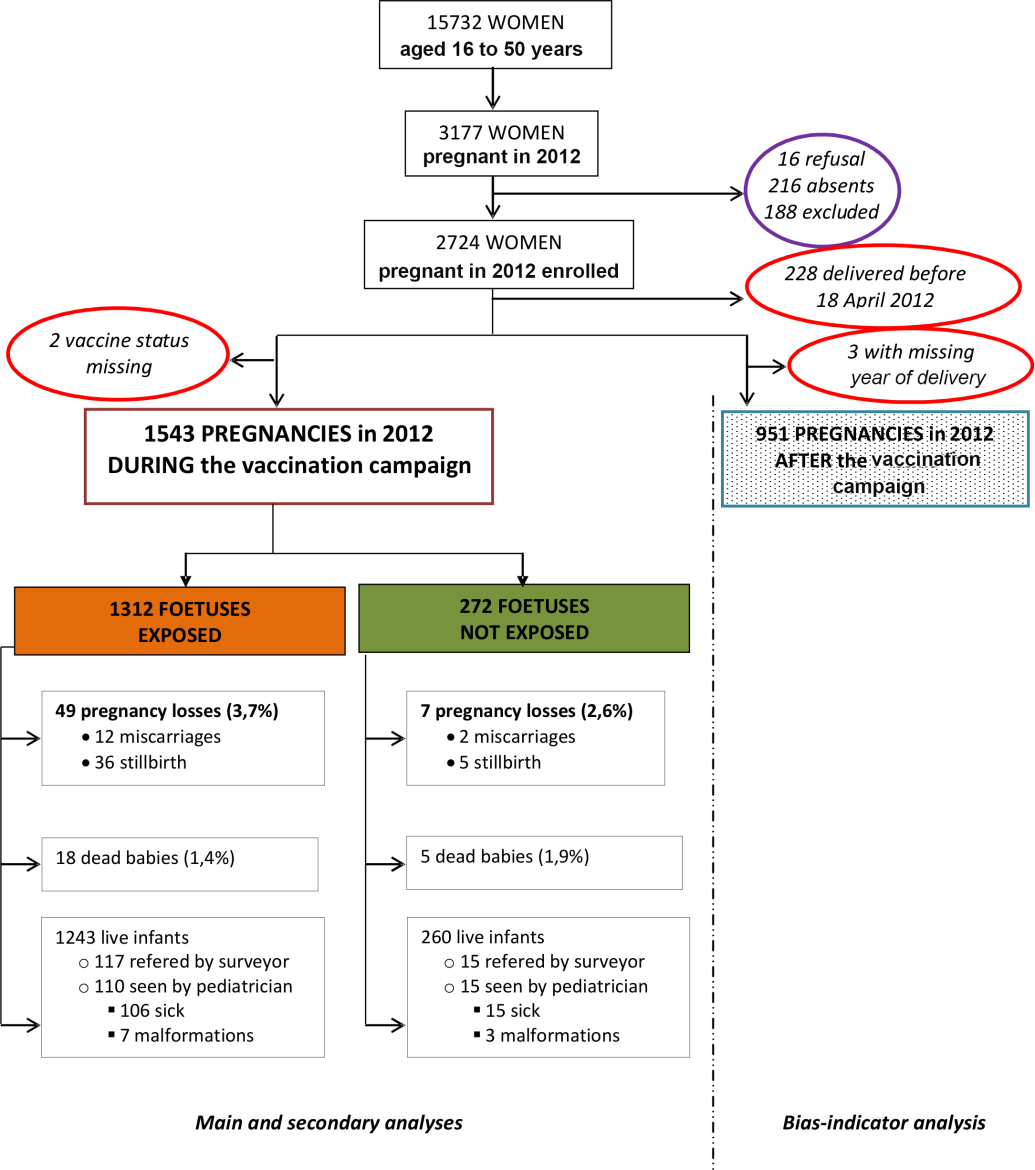
Enrollment of study participants for the primary analysis and the bias-indicator analysis, Boffa prefecture, Guinea, 2013.

From 11 November to 4 December 2013, 10,211 households were visited; 315 were absent (3.1%) and 13 refused to participate (0.1%). A total of 15,732 women 16 to 50 years old were asked about their pregnancy status and 3,177 (20.2%) reported a pregnancy in 2012 (Fig 2). After applying the exclusion criteria, 2,724 women pregnant in 2012 were enrolled; however, 231 were excluded at the time of the analysis (Fig 2). One woman was pregnant twice in 2012. A total of 2,494 pregnancies were therefore included in the analyses; 1,543 in the primary analysis and 951 in the bias-indicator analysis.

### Baseline characteristics

Overall, 84.8% [95%CI: 83.0–86.6%] of the women pregnant during the campaign received at least one dose of OCV and could therefore have exposed their fetus to the vaccine. Vaccine coverage was significantly higher among women who were pregnant during the vaccination campaign (primary analysis) than those who became pregnant after the campaign (bias-indicator analysis), both for the first round (81.1% [95%CI: 79.2–83.1%] vs 76.1% [95%CI: 73.4–78.8%], p-value = 0.003) and the second round (64.0% [95%CI: 61.6–66.4%] vs 55.5% [95%CI: 52.4–58.7%], p-value<0.001). Vaccination status was confirmed by vaccination card in 24% of the cases.

Women vaccinated during their pregnancy were not significantly different from those not vaccinated in terms of socio-demographic variables, pregnancy history, pregnancy status and practices, aside from owning a television (p = 0.033) and an oven (p<0.001) (Table 1). Vaccinated and non-vaccinated women included in the bias-indicator analysis were also similar in their baseline characteristics (Table A in S1 Appendix).

**Table 1:** Baseline characteristics and pregnancy characteristics and practices for women pregnant during the cholera mass vaccination campaign included in the analysis, Boffa prefecture, Guinea, 2013.

		Vaccinated		Non-vaccinated		
		N	% (or SD)	N	% (or SD)	p-value
N		1,308		235		
**Mean age in years** *		26.0	(10)	25.4	(10)	0.155
**Sub-prefecture**						0.942
	Koba	822	*62*.*8*	147	*62*.*6*	
	Boffa	486	*37*.*2*	88	*37*.*5*	
**Literate (yes)**		214	*16*.*4*	42	*17*.*9*	0.475
**Educational level**						0.434
	No education	1,079	*82*.*5*	185	*78*.*7*	
	Primary school	113	*8*.*6*	21	*8*.*9*	
	Secondary school	112	*8*.*6*	29	*12*.*3*	
	University	3	*0*.*2*	0	*0*.*0*	
	Unknown	1	*0*.*1*	0	*0*.*0*	
**Occupation**						0.083
	Housewife	876	*67*.*0*	150	*63*.*8*	
	Vendor	245	*18*.*7*	47	*20*.*0*	
	Student	54	*4*.*1*	12	*5*.*1*	
	Farmer	39	*3*.*0*	6	*2*.*6*	
	Fisherman	10	*0*.*8*	0	*0*.*0*	
	Unemployed	11	*0*.*8*	3	*1*.*3*	
	Other	72	*5*.*5*	17	*7*.*2*	
	Unknown	1	*0*.*1*	0	*0*.*0*	
**Mean household size** *		9.6	(6)	10.3	(8)	0.267
**Goods owned by household**						
	Radio	901	*68*.*9*	158	*67*.*2*	0.708
	Bicycle	800	*61*.*2*	140	*59*.*6*	0.714
	Mobile phone	1,168	*89*.*3*	210	*89*.*4*	1.000
	Generator	207	*15*.*8*	49	*20*.*9*	0.070
	Television	175	*13*.*4*	44	*18*.*7*	**0**.**033**
	Refrigerator	9	*0*.*7*	3	*1*.*3*	0.575
	Oven	429	*32*.*8*	110	*46*.*8*	**<0.001**
	Boat/pirogue	115	*8*.*8*	23	*9*.*8*	0.259
**Documentation of pregnancy**		1,104	*84*.*4*	197	*83*.*8*	0.846
**Had antenatal examination**		1,288	*98*.*5*	230	*97*.*9*	0.072
**Type of pregnancy**						0.564
	Singleton	1,274	*97*.*4*	229	*97*.*5*	
	Multiple	33	*2*.*6*	5	*2*.*1*	
	Unknown	1	*0*.*1*	1	*0*.*4*	
**Trimester on 18 April 2012**						0.952
	1^st^	638	*47*.*5*	118	*49*.*2*	
	2^nd^	363	*27*.*0*	65	*27*.*1*	
	3^rd^	334	*24*.*9*	56	*23*.*3*	
	Unknown	9	*0*.*7*	1	*0*.*4*	
**Birth attendant**						0.617
	Doctor	26	*2*.*0*	3	*1*.*3*	
	Nurse/midwife	800	*61*.*2*	137	*58*.*3*	
	Traditional midwife	423	*32*.*3*	83	*35*.*3*	
	Other	35	*2*.*7*	9	*3*.*8*	
	None	3	*0*.*2*	1	*0*.*4*	
	Not applicable, miscarriage	17	*1*.*3*	2	*0*.*9*	
	Unknown	4	*0*.*3*	0	*0*.*0*	
**Place of delivery**						0.054
	Hospital	56	*4*.*3*	9	*3*.*8*	
	Health center	760	*58*.*1*	126	*53*.*6*	
	Woman’s house	298	*22*.*8*	58	*24*.*7*	
	Traditional midwife’s house	169	*12*.*9*	33	*14*.*0*	
	Other	7	*0*.*6*	5	*2*.*1*	
	Not applicable, miscarriage	17	*1*.*3*	2	*0*.*9*	
	Unknown	1	*0*.*1*	2	*0*.*9*	
**Number of children born before the pregnancy in 2012**						0.066
	0	272	*20*.*8*	57	*24*.*3*	
	1	255	*19*.*5*	57	*24*.*3*	
	2	239	*18*.*3*	32	*13*.*6*	
	3	164	*12*.*5*	32	*13*.6	
	4	140	*10*.*7*	21	*8*.*9*	
	5 and over	234	*17*.*9*	35	*14*.*9*	
	Unknown	4	*0*.*3*	1	*0*.*4*	
**Mean age in years at first pregnancy** *		16.8	(3)	17.0	(2)	0.591
**Status of the last child born**						0.462
	Alive	962	*73*.*6*	170	*72*.*3*	
	Dead	79	*6*.*0*	10	*4*.*3*	
	No previous live births	257	*19*.*7*	52	*22*.*1*	
	Unknown	10	*0*.*8*	3	*1*.*3*	
**Mean age of the last born in months** *		46.5	(12)	47.7	(12)	0.203
**Number of miscarriages before 2012**						0.748
	0	1130	*86*.*4*	209	*88*.*9*	
	1	132	*10*.*1*	20	*8*.*5*	
	2	28	*2*.*1*	5	*2*.*1*	
	3 and over	14	*1*.*1*	0	*0*.*0*	
	Missing	4	*0*.*3*	1	*0*.*4*	
**Number of stillbirths before 2012**						0.356
	0	1186	*90*.*7*	216	*91*.*9*	
	1	103	*7*.*9*	14	*6*.*0*	
	2	13	*1*.*0*	3	*1*.*3*	
	3 and over	5	*0*.*4*	1	*0*.*4*	
	Missing	1	*0*.*1*	1	*0*.*4*	
**Had episode of malaria in 2012**		850	*65*.*0*	152	*64*.*7*	0.948
**Had episode of cholera in 2012**		22	*1*.*7*	6	*2*.*6*	0.509
**At-risk behaviors**						
	Coffee consumption	106	*8*.*1*	16	*6*.*8*	0.260
	Alcohol consumption	74	*5*.*7*	18	*7*.*7*	0.215
	Drug use	12	*0*.*9*	0	*0*.*0*	0.101

* Values for these variables represent the average and the standard deviation (SD).

### Pregnancy characteristics

Most (84.3%) of the women pregnant during the vaccination campaign presented a child health record booklet. The percentage of women who received antenatal care services and who delivered in a health facility was higher among those who received the vaccine during their pregnancy than those who did not, though the differences were not statistically significant (Table 1).

### Pregnancy losses

A total of 1,584 fetuses whose mother was pregnant during the campaign were included in the primary analysis; 1,312 (82.8%) were exposed to the vaccine (Table 2). A total of 56 fetuses were classified as lost. There was no difference in the crude cumulative incidence of pregnancy loss between fetuses exposed to the vaccine and those who were not (p = 0.350).

**Table 2:** Risk of negative pregnancy outcomes and malformations for fetuses exposed to OCV (primary analysis) and fetuses not exposed but born to vaccinated women (bias-indicator analysis). Boffa prefecture, Guinea, 2013.

		Primary and secondary analyses										Bias-indicator analysis					
		Fetus exposed (n = 1312)		Fetus non-exposed (n = 272)		Unadjusted relative risk			Adjusted relative risk			Unadjusted relative risk			Adjusted relative risk		
		N	CCI% [95%CI]	N	CCI% [95%CI]	uRR	[95%CI]	p-value	aRR	[95%CI]	p-value	uRR	[95%CI]	p-value	aRR	[95%CI]	p-value
**Pregnancy loss**		**49**	**3.7 [2.7–4.8]**	**7**	**2.6 [0 7–4.5]**	**1.45**	**[0.66–3.17]**	**0.350**	**1.13** ^**a**^	**[0.54–2.38]**	**0.738**	**1.06**	**[0.36–3.14]**	**0.915**	**1.19**	**[0.47–3.00]**	**0.717**
	Miscarriage*	12	1.4 [0.6–2.2]	2	1.0 [<0 1–2.4]	1.36	[0.31–6.08]	0.689	1.29^b^	[0.29–5.79]	0.736	0.80	[0.16–3.91]	0.779	0.92	[0.19–4.38]	0.915
	Stillbirth**	36	2.8 [1.9–3.7]	5	1.9 [0 2–3.5]	1.50	[0.59–3.79]	0.394	1.39^c^	[0.57–3.38]	0.464	1.32	[0.29–5.99]	0.716	1.36	[0.34–5.42]	0.658
*Live birth*		*1263*	*/*	*265*	*/*	*/*			*/*			*/*			*/*		
	*Death*	*18*	*1*.*4 [0*.*7–2*.*1]*	*5*	*1*.*9 [0*.*2–3*.*5]*	*/*			*/*			*/*			*/*		
	*Illnessⱡ*	*107*	*/*	*15*	*/*	*/*			*/*			*/*			*/*		
	**Malformationⱡ**	**7**	**0.6 [0.1–1.0]**	**3**	**1.2 [0.0–2.5]**	**0.49**	**[0.13–1.87]**	**0.296**	**0.50** ^**d**^	**[0 13–1 91]**	**0.314**	**0.45**	**[0.11–1.86]**	**0.269**	**0.51**	**[0.13–2.02]**	**0.341**

CCI: Crude Cumulative Incidence

CI: Confidence Interval

uRR/aRR: Unadjusted/adjusted Relative Risk

* Women who had been pregnant for more than 5 months on 18 April 2012 were excluded.

** Women who did not complete 5 months of pregnancy were excluded.

ⱡ According to pediatrician’s examination

a Adjusted for subprefecture, household size, antenatal care, multiple pregnancies, number of live babies delivered, number of previous stillbirths, whether they had cholera in 2012.

b Adjusted for age, level of education, household size, previous child alive or dead, whether they had cholera in 2012.

c Adjusted for profession, household size, antenatal care, multiple pregnancies, number of previous miscarriages, number of previous stillbirths, whether they had cholera in 2012.

d Adjusted for profession, radio owned by the household, consumption of coffee during the pregnancy.

The adjusted risk ratio for pregnancy loss (aRR) was 1.13 [95%CI: 0.54–2.38, p-value = 0.738] (Table 2). The risk of pregnancy loss was found to be higher among fetuses of mothers who reported a cholera episode in 2012 than those who did not in the adjusted analysis (aRR = 3.18 [95%CI: 1.56–6.48], p-value = 0.002) (Tables B-D in S1 Appendix). The interaction between the trimester of pregnancy on April 18, 2012 and the primary exposure was not significant (p = 0.465) (Table G in S1 Appendix). In the bias-indicator analysis, the risk of pregnancy loss was not associated with the vaccination status (aRR = 1.19 [95%CI: 0.47–3.00], p-value = 0.717).

### Mortality and health status for babies born alive

A total of 1,263 fetuses exposed to the vaccine and 265 non-exposed fetuses were born alive. Among them, 18 exposed (1.4% [95%CI: 0.7–2.1%]) and five non-exposed (1.9% [95%CI: 0.2–3.5%]) babies died before the survey. This difference was not statistically significant (p-value = 0.577). In addition, 133 children (8.8%) were referred to the study pediatrician among those screened in the primary analysis, as were 87 children (9.4%) in the bias-indicator study.

After the pediatrician’s clinical examination, seven vaccine-exposed children and three non-exposed children were considered to have a malformation (Table 2). Malformations were mainly from limbs (five from lower limbs and two from hands) (Table E in S1 Appendix). There was no statistically significant increase in the risk of malformation for fetuses exposed to OCV in the primary analysis (p-value = 0.314) (Table 2). After adjusting for other factors, the risk of malformation was significantly associated with the mother’s profession (p-value = 0.008) (Table F in S1 Appendix). In the bias-indicator analysis, the risk of malformation was not associated with vaccination status (aRR = 0.51 [95%CI: 0.13–2.02], p-value = 0.341).

## Discussion

These are the first estimates of the risk of pregnancy loss following vaccination of pregnant women with the bivalent, whole-cell only oral cholera vaccine. Exposure of the fetus to this vaccine was not significantly associated with the risk of pregnancy loss and malformation in this study. Vaccine coverage among pregnant women was high (83%) and similar to the overall vaccination coverage of the campaign [11]. This suggests that pregnant women who were offered OCV during the campaign chose to participate rather than forego vaccination. Vaccination coverage was higher among women who were pregnant during the campaign than among those who become pregnant after the campaign. Pregnant women may have been better informed about the vaccination campaign, less occupied by outside activities on the day of vaccination, and more willing to follow the advice of the Ministry of Health to get the vaccination than non-pregnant women. Overall, vaccinated and non-vaccinated women had similar baseline characteristics, both in the primary and in the bias-indicator analyses. Vaccinated pregnant women included in the primary analysis were more likely to attend antenatal care services and delivered more frequently in health facilities than those not vaccinated, which could be the result of a greater interest and awareness of preventive activities during pregnancy.

The lack of association between the exposure of the fetus to OCV and pregnancy loss in both the crude and the adjusted primary analysis is consistent with the findings with Dukoral in Zanzibar (aRR = 1.62 [0.76–3.43], p-value = 0.21) [9]. In the present study, the exposure of the fetus to OCV was not significantly associated with miscarriage or stillbirth. In the Zanzibar study, analysis of pregnancy loss was not broken down by miscarriage or stillbirth, although the crude incidence of stillbirths was slightly higher among vaccinated women (4.6% *versus* 2.1%) [9].

Another key finding in this study is that women who reported having had cholera in 2012 while they were pregnant were at six times higher risk of miscarriage and three times higher risk of having a stillborn child than women who did not report having had cholera. Although consistent with the literature [1–7], biological confirmation of cholera cases and determination of the date of onset of the illness would have strengthened the causal link between cholera episodes and pregnancy loss. The number of reported cholera episodes was lower among vaccinated versus non-vaccinated women who were pregnant during the campaign. This is in line with the vaccine effectiveness (86%) reported following the campaigns in Guinea [17].

The main reason newborns were referred to the pediatrician for clinical examination was illness rather than malformation. Malformations were detected mainly on upper and lower limbs. After adjusting on other factors, exposure to OCV was not statistically associated with malformation.

There are several important limitations of note in this study. First, the incidence of pregnancy loss was lower than expected both in vaccinated and non-vaccinated women, especially in the first trimester. Pregnant women may not have reported, or been aware of, pregnancy losses during the study period. Conversely, some women could have falsely reported pregnancies or loss of pregnancies, since few pregnancy losses could be verified on official documentation. Since the number of pregnancy losses is low, this possible information bias could affect our point estimates, though it is difficult to determine in which direction. Second, less than 25% of the women could present a vaccination card, leading to potential misclassification of their vaccination status. In order to minimize this potential bias, we reminded participants about the way the vaccination campaigns were organized and the route of administration. To understand further the potential presence of information bias, we conducted a bias-indicator analysis to estimate the risk of pregnancy loss among women who were pregnant after the vaccination campaign. As in the primary analysis, the risk of pregnancy loss in the bias-indicator analysis was slightly but not significantly higher among vaccinated women.

Another possible bias influencing our results is the presence of a seasonal component in pregnancies and pregnancy losses (Fig A in S1 Appendix). When comparing non-vaccinated women, the incidence of pregnancy loss was higher among women who were pregnant during the campaign than among women who become pregnant afterwards. We could therefore not consider fetuses conceived after the vaccination campaign as controls in the primary analysis, reducing the power of our study.

Lastly, as previously discussed, the number of negative events was lower than expected and the vaccine coverage was higher than expected, leading to a low number of non-exposed fetuses with negative events. This reduced the power of our analysis to detect statistical differences.

In conclusion, we found no association between fetal exposure to OCV and risk of pregnancy loss or malformation. Despite the weaknesses of a retrospective design and a decreased statistical power due to the low number of fetuses not exposed to the vaccine, we can conclude that if there is a risk of poor pregnancy outcomes from taking OCV during pregnancy, it is likely to be very small. Further studies are needed to confirm these results and provide further evidence about the risks and benefits of OCV for pregnant women and their fetus. As far as possible, these studies should be prospective cohort studies to reduce the likelihood of misclassifying negative pregnancy outcomes or exposure to the vaccine.

It is also important to note that any small potential risk of pregnancy loss could be offset by the possible benefit of vaccination. During preventive campaigns in non-epidemic periods, if the risk of infection is low, vaccination of pregnant women could be delayed, notably for women who have other risk factors for pregnancy loss. However, during epidemics, when the risk of cholera infection is high, vaccination should be offered to all pregnant women, since they are at particularly high risk of losing their fetus if they become ill with cholera.

## Supporting Information

S1 Checklist STROBE checklist. (DOC)

S1 Appendix Supplementary information. (DOC)
