# Supplementary material for: Pregnancy Outcomes after a Mass Vaccination Campaign with an Oral Cholera Vaccine in Guinea: A Retrospective Cohort Study
Source: PLoS Negl Trop Dis. 2015 Dec 29;9(12):e0004274. doi: 10.1371/journal.pntd.0004274 (PMC4695076; doi:10.1371/journal.pntd.0004274)
Supplement: S1 Checklist — (DOC) [file pntd.0004274.s001.doc]

STROBE Statement—Checklist of items that should be included in reports of ***cohort studies***

|  | Item No | Recommendation | Page | Citation |
| --- | --- | --- | --- | --- |
| **Title and abstract** | 1 | (*a*) Indicate the study’s design with a commonly used term in the title or the abstract | 1 | In the tile, “a **retrospective cohort study**” |
|  |  | (*b*) Provide in the abstract an informative and balanced summary of what was done and what was found | 2 | “From 11 November to 4 December 2013, we conducted an exhaustive retrospective cohort study in Boffa prefecture” and then “A total of 2494 pregnancies were included in the analysis. The crude incidence of pregnancy loss was 3.7% (95%CI 2.7-4.8) for foetuses exposed to BivWC and 2.6% (0.7-4.5) for non-exposed foetuses.” |
| Introduction | | |  |  |
| Background/rationale | 2 | Explain the scientific background and rationale for the investigation being reported | 3-4 | “Cholera presents a risk of complications for pregnant women and their foetus.”, “However, pregnant women have been excluded systematically as part of the target population from prior mass vaccination campaigns because of absence of strong evidence of safety of Dukoral during preganacy.[10] There is no information on the safety of BivWC for pregnant women.”, “understanding the safety of BivWC during pregnancy would provide essential information for its use in the future.” |
| Objectives | 3 | State specific objectives, including any prespecified hypotheses | 4 | “**In order to assess whether there was a difference in pregnancy outcomes between women who exposed their fetus to the OCV and those who did not**, we report the results of a retrospective cohort, which compared incidence of pregnancy losses (miscarriage and stillbirth) and malformations between these two groups.“ |
| Methods | | |  |  |
| Study design | 4 | Present key elements of study design early in the paper | 5 | “The **exhaustive retrospective cohort study** was conducted in two of these subprefectures” |
| Setting | 5 | Describe the setting, locations, and relevant dates, including periods of recruitment, exposure, follow-up, and data collection | 5 and 9 | “The study took place **in Boffa Prefecture, Republic of Guinea** where six subprefectures bordering the ocean were targeted by the **mass vaccination campaigns**. All residents above one year of age were offered a **first dose from 18 to 23 April and a second dose from 9 to 14 May 2012**” and “From 11 November to 4 December 2013, 10211 households were visited” |
| Participants | 6 | (*a*) Give the eligibility criteria, and the sources and methods of selection of participants. Describe methods of follow-up | 5-6 | “Women were included in the study if they were residents of the Koba and Boffa subprefectures, were 15 to 49 years old, pregnant in 2012 (i.e. conception and/or birth occurred in 2012) and if they (or their guardians for minor participants) provided informed consent. Exclusion criteria were non-residence in Boffa prefecture at the time of mass vaccination campaign, absence after two surveyor’s visits, did not know their vaccination status or refused to participate.“, “[The surveyors] visited all households (…)asked the head of household for the number of women aged between 16 and 50 years living in the household, and among them the number of women who were pregnant in 2012, irrespective of pregnancy outcome. The surveyors obtained written informed consent from the women who were pregnant in 2012 and conducted face-to-face interviews in the local language.” |
|  |  | (*b*) For matched studies, give matching criteria and number of exposed and unexposed |  | Non applicable |
| Variables | 7 | Clearly define all outcomes, exposures, predictors, potential confounders, and effect modifiers. Give diagnostic criteria, if applicable | 6 | “The **main outcome** was the incidence of pregnancy loss, defined as any loss of a product of conception after the woman recognises she is pregnant. Secondary outcomes included the incidence of miscarriage, stillbirth and malformation in live children. A miscarriage was defined as a loss of a clinically recognized pregnancy before the sixth month of gestation; a stillbirth as the delivery of a dead fetus (without pulse) after the fifth month of gestation. (…) A malformation was defined as physical defect reportedly identified on a living baby by the pediatrician.”  **“Primary exposure** was the intake of the OCV during pregnancy. (…) A foetus was considered as exposed if the mother was pregnant during the campaign, received at least one dose of OCV (card-confirmed or orally reported) and if at least one dose was received after date of conception and before date of birth/loss.” |
| Data sources/ measurement | 8* | For each variable of interest, give sources of data and details of methods of assessment (measurement). Describe comparability of assessment methods if there is more than one group | 6 | “A **standardized pre-piloted questionnaire** was used to collect inclusion criteria, socio-demographic data, information about the pregnancy, obstetric antecedents and other risk factors for pregnancy loss. **Vaccination status was assessed at the end of the questionnaire.”**  “Mothers and children were referred to a paediatrician if the questionnaire elicited concerns. The **pediatrician completed a clinical examination** and determined if the child was ill or presented any malformation.”  “**These outcomes** [pregnancy losses, miscarriages and stillbirth] **were orally reported** by the mother and verified on documentation when possible. A malformation was defined as physical defect reportedly identified on a living baby by the pediatrician.”  “Participants were asked whether they had been vaccinated and, if so, to show their vaccination cards. (…) Date of birth was orally reported and verified on documentation when possible; date of conception was calculated by subtracting the duration of the pregnancy (orally reported or confirmed by documentation) to the date of birth/loss. When date of birth/loss was unknown, the mother was asked if she was pregnant during the vaccination campaign.” |
| Bias | 9 | Describe any efforts to address potential sources of bias | 7 | “The same approach was applied to fetus conceived in 2012 but after the second round of vaccination, as a **bias-indicator analysis**.” |
| Study size | 10 | Explain how the study size was arrived at | 5 | “We assumed a 10% incidence of pregnancy loss, a ratio of unexposed/exposed of 0·3 (77% of pregnant women vaccinated in the vaccination coverage survey), an alpha error of 0.05, and a statistical power of 0·8, 1200 vaccinated pregnant women and 360 unvaccinated pregnant women were necessary to estimate a 1·5 increase in the risk of pregnancy loss among vaccinated women.“ |
| Quantitative variables | 11 | Explain how quantitative variables were handled in the analyses. If applicable, describe which groupings were chosen and why | 7 | “Qualitative and quantitative variables were compared respectively through Fisher and Wilcoxon tests.” |
| Statistical methods | 12 | (*a*) Describe all statistical methods, including those used to control for confounding | 8 | “Primary data analysis was based on women pregnant during the mass vaccination campaign. Descriptive analysis was stratified by vaccination status. Qualitative and quantitative variables were compared respectively through Fisher and Wilcoxon tests. The fetus was then considered as the statistical unit as some women had multiple pregnancies. We calculated crude cumulated incidence of pregnancy loss as the number of pregnancy losses divided by the number of conceived fetuses. We compared the risk of pregnancy loss through a binomial regression. The possible confounders were variables for which p-values were less than 0·20 in the bivariate analysis. We obtained an adjusted estimate of relative risk (aRR) of pregnancy loss and its 95% confidence interval (95%IC) according to OCV exposure using a forward stepwise procedure. All covariates significantly associated with the risk of a pregnancy loss (p-value<0·05) or those improving model fit (assessment based on the BIC indicator) were retained in the final model.  In a secondary analysis, the same procedure was applied to other negative outcomes (miscarriage, stillbirth and malformations). Fetuses born from mothers who had been pregnant for more than 5 months on 18 April 2012 were excluded from the analysis of the risk of miscarriage; fetuses who did not complete 5 months of gestation were excluded from the analysis of risk of stillbirth; children who were not alive at the time of the survey (foetal or perinatal deaths) were excluded from the analysis of the risk of malformations.“ |
|  |  | (*b*) Describe any methods used to examine subgroups and interactions | 7 | “The interaction between months of pregnancy on 18 April 2012 and primary exposure was tested.” |
|  |  | (*c*) Explain how missing data were addressed |  | “Women with missing data were excluded from the analysis.” |
|  |  | (*d*) If applicable, explain how loss to follow-up was addressed |  | Not applicable |
|  |  | (*e*) Describe any sensitivity analyses | 8 | “Finally, we conducted sensitivity analyses by excluding the fetus born/loss in the seven days following the first round of the mass vaccination campaign and those whom date of conception was estimated in the two weeks following the first round of the vaccination campaign.“ |
| Results | | |  |  |
| Participants | 13* | (a) Report numbers of individuals at each stage of study—eg numbers potentially eligible, examined for eligibility, confirmed eligible, included in the study, completing follow-up, and analysed | 9 | “From 11 November to 4 December 2013, 10211 households were visited; 315 were absent (3.1%) and 13 refused to participate (0.1%); 15732 women aged 16 to 50 years were asked about their pregnancy status and 3177 (20.2%) reported a pregnancy in 2012 (Figure 2). A total of 2724 women pregnant in 2012 were enrolled after applying the exclusion criteria, but 231 were excluded at the time of the analyses. One woman was pregnant twice in 2012. A total of 2494 pregnancies were therefore included in the analyses, 1543 in the primary analysis and 951 in the bias-indicator analysis” |
|  |  | (b) Give reasons for non-participation at each stage | 9 | idem |
|  |  | (c) Consider use of a flow diagram | 9 | “(Figure 2)” |
| Descriptive data | 14* | (a) Give characteristics of study participants (eg demographic, clinical, social) and information on exposures and potential confounders | 9 | “Women vaccinated during pregnancy were not significantly different from those not vaccinated, aside from owing a television (p=0.033) and an oven (p<0.001) (Table 1). Women included in the bias-indicator analysis were also similar in the baseline characteristics (eTable 1).” |
|  |  | (b) Indicate number of participants with missing data for each variable of interest | 9 | “(Figure 2)” |
|  |  | (c) Summarise follow-up time (eg, average and total amount) |  | Not applicable |
| Outcome data | 15* | Report numbers of outcome events or summary measures over time | 10 | “A total of 56 fetuses were declared as lost.” And “After pediatrician’s clinical examination, seven exposed children and three non-exposed children were considered as presenting a malformation” |
| Main results | 16 | (*a*) Give unadjusted estimates and, if applicable, confounder-adjusted estimates and their precision (eg, 95% confidence interval). Make clear which confounders were adjusted for and why they were included | 10 | “There was no difference in the crude cumulative incidence of pregnancy loss (p=0.350) (Table 2).  The adjusted risk ratio (aRR) was 1.13 [95%CI: 0.54-2.38, p-value=0.738]. After adjusting on other factors, the risk of pregnancy loss was higher for fetuses whom the mother declared an episode of cholera in 2012 (aRR=3.18 [95%CI: 1.56-6.48], p-value=0.002) (eTables 2 to 4).“  And “There was no statistically significant increase in the risk of malformation for fetuses exposed to the OCV in the primary analysis (p-value=0.314) (Table 2). After adjusting on other factors, the risk of malformation was significantly associated with mother’s profession (p-value=0.008) (eTable 6).” |
|  |  | (*b*) Report category boundaries when continuous variables were categorized |  | Not applicable |
|  |  | (*c*) If relevant, consider translating estimates of relative risk into absolute risk for a meaningful time period |  | Not applicable |
| Other analyses | 17 | Report other analyses done—eg analyses of subgroups and interactions, and sensitivity analyses | 10 | “The interaction between the months of pregnancy on April 18, 2012 and the primary was not significant (p=0.448). In the bias-indicator analysis, the risk of pregnancy losses was not associated with the vaccination status (aRR=1.19 [95%CI: 0.47-3.00], p-value=0.717).” |
| Discussion | | |  |  |
| Key results | 18 | Summarise key results with reference to study objectives | 11 | “Exposure to OCV was not significantly associated with the risk of pregnancy loss and malformation in this study.” |
| Limitations | 19 | Discuss limitations of the study, taking into account sources of potential bias or imprecision. Discuss both direction and magnitude of any potential bias | 12 | “There are **several important limitations** of note. First, women may not have reported, or been aware of, pregnancy losses in 2012. Conversely, some women could have falsely reported pregnancies or loss of pregnancies as few pregnancy losses could be verified on official documentation. As the number of pregnancy losses is low, this declaration bias could potentially affect our point estimates, being difficult to determine in which direction. Second, less than 25% of the women could show a vaccination card, leading to potential misclassification. In order to minimize this potential bias, we reminded participants about the way the vaccination campaigns were organized and the route of administration. Nevertheless, the incidence of stillbirth was higher among women who could show a card to confirm they were vaccinated than among the women who orally reported to be vaccinated. This information bias could therefore have diluted the risk of stillbirth among exposed fetuses. To understand further the potential presence of information bias, we conducted a supplementary bias-indicator analysis estimating the risk of pregnancy loss among women who were pregnant after the vaccination campaign. The risk was not significantly different between vaccinated and unvaccinated women. Pregnancy losses, as birth, might have a seasonal component. When comparing unvaccinated women, the incidence of pregnancy loss is higher among women who were pregnant during the campaign than among women pregnant after. We could therefore not consider fetus conceived after the vaccination campaign as controls, reducing the power of our analysis. Lastly, the number of negative events was low and the vaccine coverage was higher than expected, leading to a low number of non-exposed fetuses. This issue reduced the power of our analysis to detect statistical differences.” |
| Interpretation | 20 | Give a cautious overall interpretation of results considering objectives, limitations, multiplicity of analyses, results from similar studies, and other relevant evidence | 13 | “In this large retrospective cohort, we found no association between fetal exposure to OCV and risk of pregnancy loss and malformation. Despite the weaknesses of a retrospective design, we can conclude that if a risk exists, it might be very low.” |
| Generalisability | 21 | Discuss the generalisability (external validity) of the study results | 13 | “Further studies are needed to contrast these results and provide further evidence about the risks and benefits of OCV for pregnant women and their fetus. As far as possible, these studies should be prospective cohorts to minimize classification bias for negative outcomes and exposure to the vaccine.” |
| Other information | | |  |  |
| Funding | 22 | Give the source of funding and the role of the funders for the present study and, if applicable, for the original study on which the present article is based | 14 | “This study was supported by Médecins sans Frontières.” And “Médecins Sans Frontières had no role in the design and conduct of the study; collection, management, analysis of the data; and the preparation of the manuscript. Médecins Sans Frontières was involved in the interpretation of the data, in the review of the manuscript; and the decision to submit the manuscript for publication.” |

*Give information separately for exposed and unexposed groups.

**Note:** An Explanation and Elaboration article discusses each checklist item and gives methodological background and published examples of transparent reporting. The STROBE checklist is best used in conjunction with this article (freely available on the Web sites of PLoS Medicine at http://www.plosmedicine.org/, Annals of Internal Medicine at http://www.annals.org/, and Epidemiology at http://www.epidem.com/). Information on the STROBE Initiative is available at http://www.strobe-statement.org.
