# Supplementary material for: Pregnancy Outcomes after a Mass Vaccination Campaign with an Oral Cholera Vaccine in Guinea: A Retrospective Cohort Study
Source: PLoS Negl Trop Dis. 2015 Dec 29;9(12):e0004274. doi: 10.1371/journal.pntd.0004274 (PMC4695076; doi:10.1371/journal.pntd.0004274)
Supplement: S1 Appendix — (DOC) [file pntd.0004274.s002.doc]

**S1. SUPPLEMENTARY INFORMATION**

Table of Contents

[S1. Description of the women included in the bias-indicator analysis, according to their vaccine status 2](#__RefHeading___Toc435738355)

[S2. Primary, secondary and bias-indicator analyses results: risk of pregnancy losses, miscarriage and stillbirth. 5](#__RefHeading___Toc435738356)

[S3. Primary, secondary and bias-indicator analyses results: risk of malformations 8](#__RefHeading___Toc435738357)

[S4. Seasonality 10](#__RefHeading___Toc435738358)

[S5. Interaction between exposure to OCV and pregnancy trimester 12](#__RefHeading___Toc435738359)

[S6. Possible information biases 13](#__RefHeading___Toc435738360)

[S7. Mortality 15](#__RefHeading___Toc435738361)

[S8: Structure of the cohort on 18th April 2012 16](#__RefHeading___Toc435738362)

# S1. Description of the women included in the bias-indicator analysis, according to their vaccine status

The Table A shows the socio-demographic characteristics, pregnancy characteristics, obstetric antecedents and other risk factors for pregnancy loss of the women included in the bias-indicator analysis.

Women were included in the bias-indicator analysis if they were residents of the Koba and Boffa subprefectures, were 15 to 49 years old, pregnant in 2012 (i.e. conception and/or birth occurred in 2012) but after the vaccination campaign and if they (or their guardians for minor participants) provided informed consent. Exclusion criteria were non-residence in Boffa prefecture at the time of mass vaccination campaign, absence after two interviewer’s visits, did not know their vaccination status or refused to participate.

In this analysis, women vaccinated were similar to those non-vaccinated (Table A). There were some significant differences between the two groups. The vaccinated women were older (p=0·039), more often housewives (p=0.017), owed more often an oven (p=0.013), reported more frequently malaria and cholera episodes in 2012 (p=0.040 and 0.034 respectively) and their last born were more frequently alive (p=0.008) than the non-vaccinated.

**Table A: Baseline characteristics for women who become pregnant after the cholera mass vaccination campaign included in the bias-indicator analysis, according to their vaccine status, Boffa prefecture, Guinea, 2013.**

|  |  | **Vaccinated** | |  | **Non-vaccinated** | |  |  |
| --- | --- | --- | --- | --- | --- | --- | --- | --- |
|  |  | **N** | **% (or SD)** |  | **N** | **% (or SD)** |  | **p-value** |
| N |  | 749 |  |  | 202 |  |  |  |
| **Mean Age in years*** | | 26.3 | (9) |  | 25.4 | (10) |  | **0**.**039** |
| **Sub-prefecture** | |  |  |  |  |  |  | 0.808 |
|  | Koba | 454 | *60.6* |  | 120 | *59.4* |  |  |
|  | Boffa | 295 | *39*.*4* |  | 82 | *40*.*6* |  |  |
| **Literate (yes)** | | 115 | *15*.*4* |  | 37 | *18.3* |  | 0.585 |
| **Educational level** | |  |  |  |  |  |  | 0.201 |
|  | No education | 624 | *83*.*3* |  | 157 | *77*.*7* |  |  |
|  | Primary school | 69 | *9*.*2* |  | 24 | *11.9* |  |  |
|  | Secondary school | 56 | *7*.*5* |  | 21 | *10*.*4* |  |  |
|  | University | 0 | *0*.*0* |  | 0 | *0*.*0* |  |  |
|  | Unknown | 0 | *0*.*0* |  | 0 | *0*.*0* |  |  |
| **Occupation** | |  |  |  |  |  |  | **0**.**017** |
|  | Housewife | 514 | *68.6* |  | 132 | *65.4* |  |  |
|  | Vendor | 30 | *4*.*0* |  | 1 | *0*.*5* |  |  |
|  | Student | 23 | *3*.*1* |  | 6 | *3*.*0* |  |  |
|  | Farmer | 119 | *15.9* |  | 35 | *17.3* |  |  |
|  | Fisherman | 0 | *0*.*0* |  | 0 | *0*.*0* |  |  |
|  | Unemployed | 6 | *0*.*8* |  | 0 | *0*.*0* |  |  |
|  | Other | 7 | *0*.*9* |  | 2 | *1*.*0* |  |  |
|  | Unknown | 4 | *0*.*5* |  | 1 | *0*.*5* |  |  |
|  | Housewife | 45 | *6*.*0* |  | 24 | *11.9* |  |  |
|  | Vendor | 1 | *0*.*1* |  | 1 | *0*.*5* |  |  |
| **Mean Household size*** | | 9.9 | (7) |  | 9.6 | (7) |  | 0.714 |
| **Goods owned by household** | |  |  |  |  |  |  |  |
|  | Radio | 495 | *66.1* |  | 135 | *66*.*8* |  | 0.967 |
|  | Bicycle | 465 | *62*.*1* |  | 109 | *54*.*0* |  | 0.091 |
|  | Mobile phone | 663 | *88*.*5* |  | 175 | *86*.*6* |  | 0.575 |
|  | Generator | 85 | *11*.*4* |  | 21 | *10*.*4* |  | 0.844 |
|  | Television | 69 | *9*.*2* |  | 18 | *8*.*9* |  | 1.000 |
|  | Refrigerator | 4 | *0*.*5* |  | 2 | *1*.*0* |  | 0.321 |
|  | Oven | 249 | *33.2* |  | 88 | *43.6* |  | **0**.**013** |
|  | Boat/pirogue | 65 | *8*.*7* |  | 19 | *9*.*4* |  | 0.827 |
| **Documentation for pregnancy** | | 667 | *89.1* |  | 177 | *87.6* |  | 0.409 |
| **Had antenatal examination** | | 734 | *98*.*0* |  | 1906 | *97*.*0* |  | 0.483 |
| **Type of pregnancy** | |  |  |  |  |  |  | 0.322 |
|  | Singleton | 728 | *97*.*2* |  | 200 | *99*.*0* |  |  |
|  | Multiple | 20 | *2*.*7* |  | 2 | *1*.*0* |  |  |
|  | Unknown | 1 | *0*.*1* |  | 0 | *0*.*0* |  |  |
| **Birth attendant** | |  |  |  |  |  |  | 0.108 |
|  | Doctor | 10 | *1*.*3* |  | 6 | *3*.*0* |  |  |
|  | Nurse/midwife | 478 | *63.8* |  | 111 | *5*.*0* |  |  |
|  | Traditional midwife | 232 | *30*.*9* |  | 64 | *36*.*6* |  |  |
|  | Other | 19 | *2*.*5* |  | 5 | *2*.*5* |  |  |
|  | None | 2 | *0*.*3* |  | 2 | *1*.*0* |  |  |
|  | Not applicable, miscarriage | 7 | *0*.*9* |  | 3 | *1*.*5* |  |  |
|  | Unknown | 1 | *0*.*1* |  | 1 | *0*.*5* |  |  |
| **Place of delivery** | |  |  |  |  |  |  | 0.146 |
|  | Hospital | 31 | *4*.*1* |  | 10 | *5*.*0* |  |  |
|  | Health center | 449 | *60.0* |  | 109 | *54.0* |  |  |
|  | At the woman’s house | 164 | *21*.*9* |  | 40 | *19*.*8* |  |  |
|  | At the traditional midwife’s | 92 | *12*.*3* |  | 39 | *19*.*3* |  |  |
|  | Other | 6 | *0*.*8* |  | 1 | *0*.*5* |  |  |
|  | Not applicable, miscarriage | 7 | *0*.*9* |  | 3 | *1*.*5* |  |  |
|  | Unknown | 0 | *0*.*0* |  | 0 | *0*.*0* |  |  |
| **Number of children born before the pregnancy in 2012** | |  |  |  |  |  |  | 0.051 |
|  | 0 | 122 | *16.3* |  | 51 | *25.3* |  |  |
|  | 1 | 151 | *20*.*2* |  | 40 | *19*.*8* |  |  |
|  | 2 | 128 | *17*.*1* |  | 27 | *13*.*4* |  |  |
|  | 3 | 96 | *12*.*8* |  | 21 | *10*.*4* |  |  |
|  | 4 | 69 | *9*.*2* |  | 23 | *11*.*4* |  |  |
|  | 5 and over | 182 | *24.3* |  | 39 | *19*.*3* |  |  |
|  | Unknown | 1 | *0*.*1* |  | 1 | *0*.*5* |  |  |
| **Mean age at the first pregnancy in years*** | | 16.7 | (3) |  | 16.6 | (3) |  | 0.143 |
| **Status of the last born baby** | |  |  |  |  |  |  | **0**.**008** |
|  | Alive | 584 | *78*.*0* |  | 136 | *67*.*3* |  |  |
|  | Dead | 46 | *6*.*1* |  | 16 | *7*.*9* |  |  |
|  | No previous live born babies | 115 | *15.4* |  | 50 | *24.8* |  |  |
|  | Unknown | 4 | *0*.*5* |  | 0 | *0*.*0* |  |  |
| **Mean age of the last born babyin months*** | | 43.4 | (12) |  | 43.7 | (12) |  | 0.916 |
| **Number of abortions before 2012** | |  |  |  |  |  |  | 0.128 |
|  | 0 | 657 | *87*.*7* |  | 171 | *64*.*7* |  |  |
|  | 1 | 71 | *9*.*5* |  | 21 | *10*.*4* |  |  |
|  | 2 | 18 | *2*.*4* |  | 6 | *3*.*0* |  |  |
|  | 3 and over | 3 | *0*.*4* |  | 4 | *2*.*0* |  |  |
| **Had episode of malaria in 2012** | | 501 | *66.9* |  | 125 | *61.9* |  | **0**.**040** |
| **Had episode of cholera in 2012** | | 14 | *1*.*9* |  | 8 | *4*.*0* |  | **0**.**034** |
| **At-risk behaviors** | |  |  |  |  |  |  |  |
|  | Coffee consumption | 73 | *9.8* |  | 19 | *9*.*4* |  | 1.000 |
|  | Alcohol consumption | 48 | *6*.*4* |  | 43 | *6*.*4* |  | 1.000 |
|  | Drug use | 9 | *1*.*2* |  | 2 | *1*.*0* |  | 1.000 |

* Values for these variables represent the average and the standard deviation (SD).

# S2. Primary, secondary and bias-indicator analyses results: risk of pregnancy losses, miscarriage and stillbirth.

- **Primary analysis**

Exposure of the fetus to OCV was not significantly associated with pregnancy loss (aRR=1.13 [95%CI: 0.54-2.38, p-value=0.738) after adjusting on other risk factors (Table B). The risk of pregnancy loss was higher among women living in Koba sub-prefecture (p=0.047), in small households (p<0. 001), did not use antenatal care services (p<0.001), had a multiple pregnancy (p<0.001), had more than 3 live born babies before 2012 (p=0.013), had already experienced stillbirth in the past (p<0.001) and declared an episode of cholera in 2012 (p=0.002).

**Table B: Adjusted relative risk of pregnancy loss for the fetuses of the women included in the retrospective cohort, Prefecture of Boffa, Republic of Guinea, 2013.**

|  |  | **PRIMARY ANALYSIS**  **(During the campaign)** | | |  | **BIAS-INDICATOR**  **(After the campaign)** | | |
| --- | --- | --- | --- | --- | --- | --- | --- | --- |
|  |  | **aRR** | **CI95%** | **p-value** |  | **aRR** | **CI95%** | **p-value** |
| **Sub-prefecture** | |  |  | **0**.**047** |  | / |  |  |
|  | Koba | 1.72 | [1.01-2.95] |  |  |  |  |  |
|  | Boffa | 1.00 | - | - |  |  |  |  |
| **Occupation** | |  |  |  |  |  |  | 0.068 |
|  | Housewife / Student / Unemployed |  |  |  |  | 1·00 | - | - |
|  | Other |  |  |  |  | 2.16 | [0.95-4.93] |  |
| **Household size** | |  |  | **<0**.**001** |  | **/** |  |  |
|  | Less 6 people | 3.76 | [1.82-7.75] | **<0**.**001** |  |  |  |  |
|  | 6 to 8 people | 1.00 | - | - |  |  |  |  |
|  | More than 8 people | 1.44 | [0.69-2.99] | 0.331 |  |  |  |  |
| **Fetus exposed to the vaccine** | | 1.13 | [0.54-2.38] | 0.738 |  | / |  |  |
| **Mother vaccinated (fetus not exposed)** | | / |  |  |  | 1.9 | [0.47-3.00] | 0.717 |
| **Use to antenatal care services** | | 0.06 | [0.03-0.11] | **<0.001** |  | 0.04 | [0.02-0.10] | **<0.001** |
| **Multiple pregnancy** | | 9.80 | [5.72-16.80] | **<0**.**001** |  | **/** |  |  |
| **Number of live born babies before 2012** | |  |  | **0**.**013** |  | **/** |  |  |
|  | Less than 2 | 1.00 | - | - |  |  |  |  |
|  | 2 or 3 | 1.77 | [0.93-3.35] | 0.081 |  |  |  |  |
|  | More than 3 | 2.31 | [1.32-4.03] | **0**.**003** |  |  |  |  |
| **Status of the last born** | | / |  |  |  |  |  | **<0**.**001** |
|  | No previous live born |  |  |  |  | 1.00 | - | - |
|  | Alive |  |  |  |  | 2.15 | [0.28-16.62] | 0.462 |
|  | Dead |  |  |  |  | 10.76 | [1.31-88.61] | 0.027 |
| **Number of stillbirth before 2012** | |  |  | **<0**·**001** |  | / |  |  |
|  | None | 1.00 | - | - |  |  |  |  |
|  | 1 | 2.37 | [1·26-4·45] | **0**·**007** |  |  |  |  |
|  | 2 and over | 4.65 | [2·66-8·12] | **<0**·**001** |  |  |  |  |
| **Episode of cholera in 2012** | | 3.18 | [1·56-6·48] | **0**·**002** |  | / |  |  |
| **At-risk behavior** | | / |  |  |  | 5.00 | [1.09-22.84] | **0**.**038** |

In the bias-indicator analysis, as expected, the vaccination was not associated with pregnancy loss (aRR=1.19 [95%CI: 0.47-3.00, p-value=0.717). The risk of pregnancy loss was higher for mothers who did not use antenatal care services (p<0.001), had her last born dead (p<0.001) and had at-risk behaviors (p=0.038).

- **Secondary analysis**

In the secondary analysis, after adjusting on other factors, the OCV intake was not significantly associated with miscarriage (aRR=1.29 [95%CI: 0.29-5.17, p-value=0.736) (Table C). The risk of miscarriage was higher among women 35 years old and above (p=0.001), educated (p<0.001), those who did not use antenatal care services (p<0.001), had a multiple pregnancy (p<0.001) and lived in small households (p=0.004).

Among the women who get pregnant after the vaccination campaign (indicator-bias analysis), and after adjusting on other factors, the vaccination was not significantly associated with miscarriage (aRR=0.92 [95%CI: 0.19-4.38, p-value=0.915) (Table C). The risk of miscarriage was higher for women who had a job (p=0.026), reported a cholera episode in 2012 (p=0.001) and those whom their last born was dead (p=0.009).

**Table C : Adjusted relative risk of miscarriage for the fet**uses of the women included in the retrospective cohort, Prefecture of Boffa, Republic of Guinea, 2013.

|  |  | **During the campaign**  **SECONDARY ANALYSIS** | | |  | **After the campaign**  **BIAS-INDICATOR** | | |
| --- | --- | --- | --- | --- | --- | --- | --- | --- |
|  |  | **aRR** | **CI95%** | **p-value** |  | **aRR** | **CI95%** | **p-value** |
| **Age in years** | |  |  | **<0**·**001** |  |  |  | 0.121 |
|  | Less than 25 | 1.43 | [0.28-7.30] | 0.666 |  | 1.00 | - | - |
|  | 25 to 34 | 1.00 | - | - |  | 2.01 | [0.24-16.49] | 0.516 |
|  | 35 and over | 13.71 | [2.91-64.54] | 0.001 |  | 7.08 | [0.87-57.59] | 0.067 |
| **Education of the mother** | | 2.61 | [2.02-3.37] | **<0**.**001** |  | / |  |  |
| **Occupation** | | / |  |  |  | 5.50 | [1.22-24.69] | **0.026** |
| **Household size** | |  |  |  |  | **/** |  |  |
|  | Less 6 people | 5.17 | [1.68-15.93] | **0**.**004** |  |  |  |  |
|  | 6 to 8 people | 1.00 | - | - |  |  |  |  |
|  | More than 8 people | 1.00 | [0.99-1.01] | 0.476 |  |  |  |  |
| **Fetus exposed to the vaccine** | | 1.29 | [0.29-5.79] | 0.736 |  | / |  |  |
| **Mother vaccinated (fetus not exposed)** | | / |  |  |  | 0.92 | [0.19-4.38] | 0.915 |
| **Status of the last born** | | / |  |  |  |  |  |  |
|  | No previous live born |  |  |  |  | 2.82 | [0.23-34.96] | 0.420 |
|  | Alive |  |  |  |  | 1.00 | - | - |
|  | Dead |  |  |  |  | 10.68 | [1.82-62.81] | **0**.**009** |
| **Episode of cholera in 2012** | | 5.87 | [0.80-43.06] | 0.082 |  | 14.15 | [2.97-67.30] | **0**.**001** |

* The women whose pregnancy began more than 5 months before 18 April 2012 were excluded of this analysis.

In the secondary analysis, after adjusting on other factors, the OCV intake was not significantly associated with stillbirth (aRR=1.39 [95%CI: 0.57-3.38, p-value=0.464) (Table D). The risk of stillbirth was higher for women 35 years older and above (p=0.001), educated women (p<0.001), those who did not look for antenatal care (p<0.001), had a multiple pregnancy (p<0.001) and lived in small households (p=0.004).

Among the women who become pregnant after the vaccination campaign (indicator-bias analysis), and after adjusting on other factors, the vaccination was not significantly associated with stillbirth (aRR=1.36 [95%CI: 0.34-5.42, p-value=0.658) (Table D). The risk of miscarriage was higher for women living in Boffa sub-prefecture (p=0.028), did not look for antenatal care (p<0.001) and reported to have a cholera episode in 2012 (p=0.006).

**Table D: Adjusted relative risk of death between 6 and 9 month of pregnancy(stillbirth) for the fet**uses of the women included in the retrospective cohort, Prefecture of Boffa, Republic of Guinea, 2013.

|  |  | **SECONDARY ANALYSIS**  **(During the campaign)** | | |  | **BIAS-INDICATOR**  **(After the campaign)** | | |
| --- | --- | --- | --- | --- | --- | --- | --- | --- |
|  |  | **aRR** | **CI95%** | **p-value** |  | **aRR** | **CI95%** | **p-value** |
| **Sub-prefecture** | | / |  |  |  |  |  | **0.028** |
|  | Koba |  |  |  |  | 1.00 | - | - |
|  | Boffa |  |  |  |  | 4.07 | [1.16-14.23] |  |
| **Districts** | | / |  |  |  |  |  |  |
|  |  |  |  |  |  | 1.00 | - | - |
|  |  |  |  |  |  | 4.93 | [1.25-19.43] | 0.023 |
|  |  |  |  |  |  | 2.41 | [0.43-15.31] | 0.315 |
| **Occupation** | |  |  | **0.013** |  | / |  |  |
|  | Housewife / Student / Unemplyed | 1.00 | - |  |  |  |  |  |
|  | Other | 2.15 | [1.17-3.95] |  |  |  |  |  |
| **Household size** | |  |  |  |  |  |  |  |
|  | Less 6 people | 4.62 | [1.94-11.01] | **0.001** |  | 1.21 | [0.25-5.88] | 0.811 |
|  | 6 to 8 people | 1.00 | - |  |  | 4.25 | [1.18-15.31] | 0.027 |
|  | More than 8 people | 1.60 | [0.67-3.78] | 0**.**289 |  | 1.00 |  |  |
| **Fetus exposed to the vaccine** | | 1.39 | [0.57-3.38] | 0**.**464 |  | / |  |  |
| **Mother vaccinated (fetus not exposed)** | | / |  |  |  | 1.36 | [0.34-5.42] | 0.658 |
| **Use of antenatal care service** | | 0.08 | [0.01-0.45] | **0.004** |  | 0.04 | [0.01-0.20] | **<0.001** |
| **Multiple pregnancy** | | 13.65 | [7.36-25.31] | **<0.001** |  | **/** |  |  |
| **Number of abortion before 2012** | |  |  | **0.055** |  | **/** |  |  |
|  | None | 1.00 | - |  |  |  |  |  |
|  | At least one | 2.02 | [0.98-4.13] |  |  |  |  |  |
| **Number of stillbirth before 2012** | |  |  |  |  | **/** |  |  |
|  | None |  |  |  |  |  |  |  |
|  | 1 | 2.76 | [1.36-5.60] | **0.005** |  |  |  |  |
|  | 2 and over | 10.56 | [3.37-33.10] | **<0.001** |  |  |  |  |
| **Episode of cholera in 2012** | | 3.28 | [1.43-7.52] | **0.005** |  | / |  |  |
| **At-risk behavior** | | / |  |  |  | 10.33 | [1.94-55.10] | **0.006** |

** The miscarriages were excluded from this analysis.*

# S3. Primary, secondary and bias-indicator analyses results: risk of malformations

Among the 1,503 live children born from mothers pregnant during the campaign recruited in the study, 133 (8.8%) were referred to the study pediatrician. Among the 925 live born babies from mothers pregnant after the campaign (indicator-bias analysis) recruited in the study, 87 (9.4%) were referred to the study pediatrician.

The babies were mainly referred because the mother declared to the interviewer that their child were sick at the time of the interview (7.3% and 7.6% of the children born from a mother pregnant respectively during and after the campaign) or that he/she suffered from of recurrent disease (1.6% and 2.5% of the children born from mothers pregnant during and after the campaign respectively).

The mother notified their child suffered from a malformation only in 1.2% and 0.9% of the children born from mothers pregnant during and after the campaign respectively.

After complete clinical examination by the study pediatrician, the incidence of malformations was 0.7% [CI 95% : 0.3-1.1] among the live born babies from women pregnant during the campaign and 0.9% [CI 95% : 0.3-1.5] among the live born babies from women pregnant after the campaign. The malformations affected mainly upper and lower limbs (Table E).

**Table E: Malformations diagnosed by the pediatrician during the examination of the children included in the retrospective cohort. Prefecture of Boffa. Republic of Guinea. 2013.**

|  |  | **During the campaign** | **After the campaign** |
| --- | --- | --- | --- |
| **Congenital cardiopathy** | | **1** | **1** |
| **Malformation of upper limbs** | | **2** | **3** |
|  | MICRODACTYLIA | 1 | 1 |
|  | BILATERAL POLYDACTYLIA | 1 | 2 |
| **Malformation of lower limbs** | | **5** | **2** |
|  | CLUB FOOT | 3 | 2* |
|  | POLYSYNDACTYLIA | 1 | 0 |
|  | POLYDACTYLIA | 1 | 0 |
| **Other malformations** | | **2** | **3** |
|  | MALFORMATION OF THE AURICLE OF THE EAR | 1 | 0 |
|  | MICROPENIS | 0 | 1 |
|  | UMBILICAL HERNIA | 1 | 2 |

* One of the club foot were not visible during the examination but has certainly existed taking into account the scare and the oral history reported by the mother.

Exposure of the fetus to OCV was not significantly associated with malformations of the live child (aRR=0.50 [95%CI: 0.13-1.91, p-value=0.314) after adjusting on other risk factors (Table F). The risk of malformation was higher for children from mother who work outside home (p=0·008).

In the bias-indicator analysis, the vaccination was not associated with malformation of the live born babies (aRR=0·51 [95%CI: 0·13-2·02, p-value=0·341). The risk of malformation was higher for babies from women who did not use the antenatal care services (p=0·001) and owed a bicycle (p=0·033).

**Table F: Adjusted relative risk of malformation for the live children whom the women included in the retrospective cohort. Prefecture of Boffa. Republic of Guinea.** 2013.

|  |  | **PRIMARY ANALYSIS**  **(During the campaign)** | | |  | **BIAS-INDICATOR**  **(After the campaign)** | | |
| --- | --- | --- | --- | --- | --- | --- | --- | --- |
|  |  | **RRa** | **IC95%** | **p-value** |  | **RRa** | **IC95%** | **p-value** |
| **Sub-prefecture** | | / |  |  |  |  |  | 0·175 |
|  | Koba |  |  |  |  | 1·00 | - | - |
|  | Boffa |  |  |  |  | 0·24 | [0·03-1·87] |  |
| **Occupation** | | 6·14 | [1·60-23·60] | **0**·**008** |  | 2·48 | [0·63-9·81] | 0·197 |
| **Radio** | | 3·95 | [0·50-30·99] | 0·191 |  |  |  |  |
| **Bicycle** | | / |  |  |  | 0·15 | [0·03-0·85] | **0**·**033** |
| **Fetus exposed to the vaccine** | | 0·50 | [0·13-1·91] | 0·314 |  | / |  |  |
| **Mother vaccinated (fetus not exposed)** | | / |  |  |  | 0·51 | [0·13-2·02] | 0·341 |
| **Use of antenatal care services** | | / |  |  |  | 0·03 | [<0·01-0·22] | **0**·**001** |
| **Episode of malaria in 2012** | | / |  |  |  | 0·22 | [0·05-1·07] | 0·061 |
| **At risk-behavior** | |  |  |  |  |  |  |  |
|  | Coffee consumption | 3·30 | [0·73-14·79] | 0·119 |  | / |  |  |

** Only the live children who were included in the analysis.*

# S4. Seasonality

The number of deliveries increased from April 2012 to October 2012, then reached a plateau until February 2013 with 190 deliveries per month on average before decreasing (Fig A).

***Fig A: Number of birth per month in 2012 and 2013, according to the vaccination status of the women included in the retrospective cohort. Boffa prefecture, Guinea, 2013.***
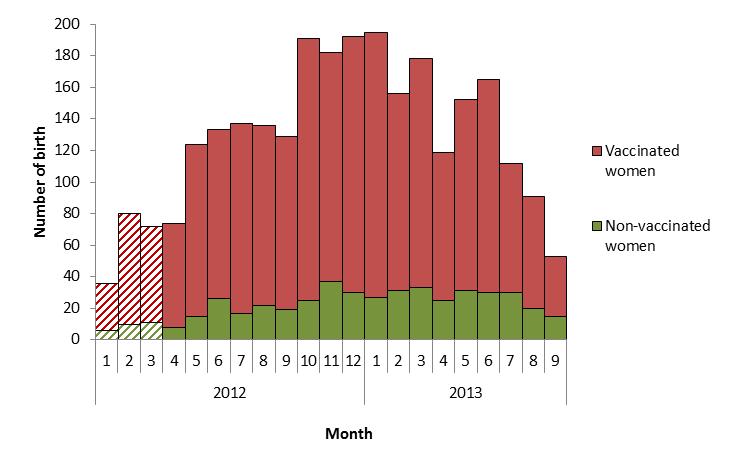


*NB: The women pregnant in 2012 but before the vaccination campaign, excluded from the analysis, were included in this graph (hatched bars).*

The vaccination occurred at different times in the course of the pregnancy. Almost half of the fetuses from pregnant women vaccinated during the campaign were exposed during the 1^st^ trimester, 28.6% during the 2^nd^ and 17.9% during the 3^rd^ trimester (Fig B). Two third of the pregnancy losses occurred between the 2nd and the 3rd month after the first dose of OCV was taken.

**Fig B: Timeline of the pregnancies with negative outcome for the women included in the primary analysis (pregnant during the vaccination campaign, Boffa prefecture, Guinea, 2013.**


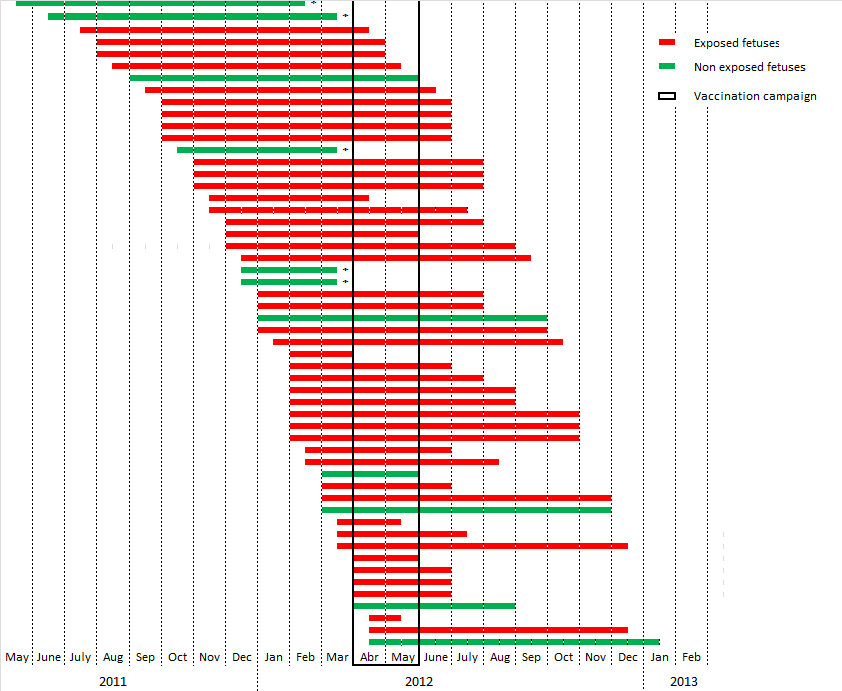


** Fetuses whom the mother was pregnant before the campaign, excluded from the analysis but included on the graph.*

*NB: Six pregnancies could not be represented of the graph since the month of conception or the month of loss were unknown.*

# S5. Interaction between exposure to OCV and pregnancy trimester

To test the hypothesis of pregnancy outcomes varying according to the time of OCV intake in the course of the pregnancy, we add an interaction term. The interaction between exposure to OCV and the trimester of pregnancy on 18 April was not significant (p=0.465) (Table G). Nevertheless, we can see a trend towards a protective effect during the first trimester and a negative effect during second and third trimester.

**Table G: Adjusted relative risk of pregnancy loss for the fetuses of the women included in primary analysis, when introducing an interaction between the exposure to the oral cholera vaccine and the trimester of exposure, Prefecture of Boffa, Republic of Guinea, 2013.**

|  |  | **PRIMARY ANALYSIS**  **(During the campaign)** | | |
| --- | --- | --- | --- | --- |
|  |  | **aRR** | **CI95%** | **p-value** |
| **Sub-prefecture** | |  |  | 0.068 |
|  | Koba | 1.67 | [0.96-2.91] |  |
|  | Boffa | 1.00 | - | - |
| **Household size** | |  |  | **<0.001** |
|  | Less 6 people | 3.45 | [1.61-7.43] | **0**. **002** |
|  | 6 to 8 people | 1.00 | - | - |
|  | More than 8 people | 1.46 | [0.68-3.15] | 0.333 |
| **Fetus exposed to the vaccine** | |  |  |  |
|  | During 1^st^ trimester | 0.75 | [0.31-1.79] | 0.515 |
|  | During 2^nd^ trimester | 2.59 | [0.34-19.61] | 0.355 |
|  | During 3^rd^ trimester | 1.65 | [0.24-11.28] | 0.611 |
| **Interaction term** | |  |  | 0.465 |
| **Antenatal examination** | | 0.05 | [0.03-0.09] | **<0.001** |
| **Multiple pregnancy** | | 10.93 | [6.16-19.40] | **<0**.**001** |
| **Number of live born babies before 2012** | |  |  | **0**.**012** |
|  | Less than 2 | 1.00 | - | - |
|  | 2 or 3 | 1.75 | [0.90-3.40] | 0.097 |
|  | More than 3 | 2.42 | [1.35-4.35] | **0**.**003** |
| **Number of stillbirth before 2012** | |  |  | **<0.001** |
|  | None | 1.00 | - | - |
|  | 1 | 2.07 | [1.04-4.11] | **0**.**038** |
|  | 2 and over | 4.91 | [2.74-8.88] | **<0.001** |
| **Episode of cholera in 2012** | | 3.13 | [1.47-6.67] | **0**.**003** |

# S6. Possible information biases

There could be two different information biases: one on the pregnancy outcome and one on the exposure to the OCV.

Concerning the possible bias in the pregnancy outcome, around 16% of the women included in the retrospective cohort could not present any official documentation proving they have really been pregnant in 2012 and less than one third of the losses were confirmed by an official documentation. Since the number of negative events was low, misclassification could have biased the estimation of the risk (Fig C). On the other hand, some women who have experienced pregnancy loss could have preferred not to notify it to the team, notably if they decided not to be vaccinated, since the interviewer wore the same “MSF” identifier than the vaccination team. This would cause an overestimation of the risk associated with the OCV intake. In our data, the incidence of pregnancy loss was higher among vaccinated women, no matter if they were pregnant during or after the vaccination campaign (Fig C).

**Fig C: Incidence of pregnancy loss, abortion and stillbirths according to the vaccination status and the time of the pregnancy, when taking into account a) all the pregnancy outcomes; or b) only the pregnancy outcomes confirmed by an official documentation.**

**
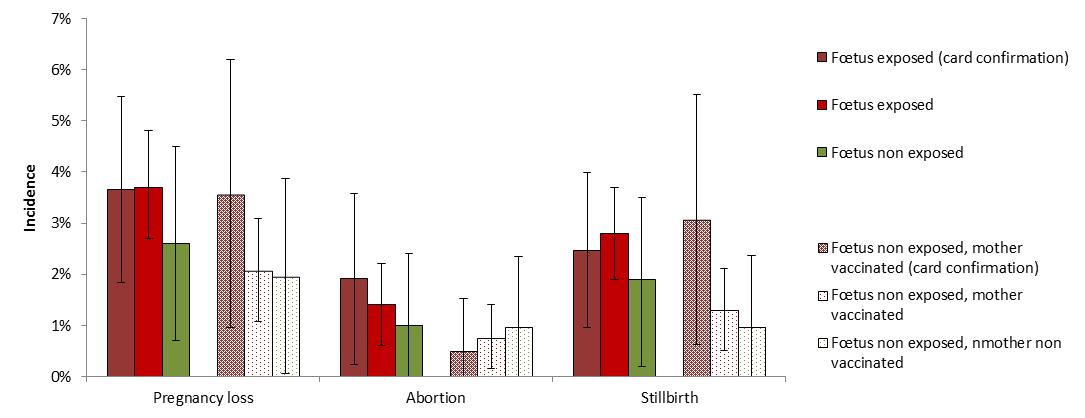
**
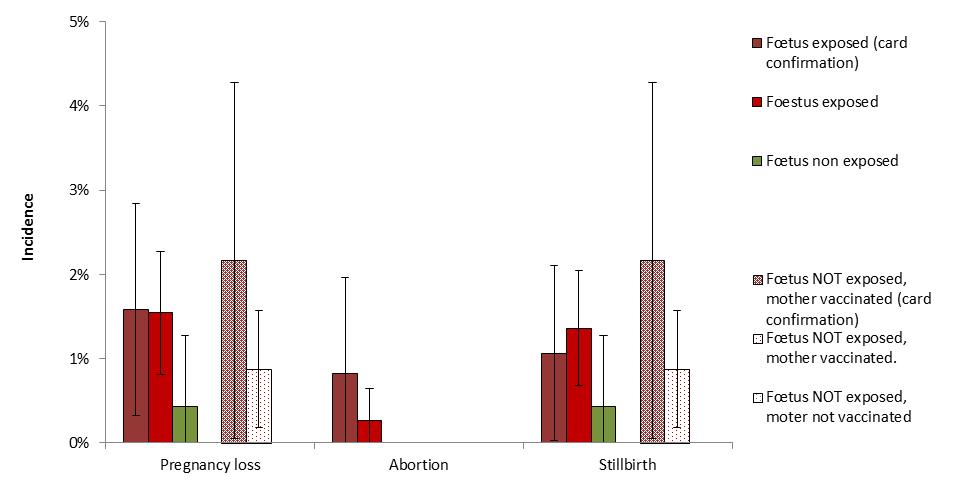


**During the campaign**

**After the campaign**

**During the campaign**

**After the campaign**

Regarding the ascertainment of the exposure to OCV, less than a quarter of the women included in the cohort could present a vaccination card. The incidence of pregnancy loss varied depending on whether vaccination status was defined by card confirmation or oral reporting (Fig C), notably for the women pregnant after the campaign. This difference was higher for the incidence of stillbirths. An information bias on vaccination status could underestimate the risk.

# S7. Mortality

The mortality of live born babies was slightly higher for the pregnancies after the campaign (Fig D). This difference was not significant, but it was not negligible considering that the follow-up was shorter for the babies born from a pregnancy post-campaign (1 to 9 month against 9 to 19 month for babies born of pregnancy that occurred respectively after and during).

The overall mortality of the fetuses (i.e. the pregnancy loss and the death of the live born baby) was at the end very similar for the pregnancies that occurred during or after the campaign.

**Fig D: Mortality of the fetuses included in the retrospective cohort, according to vaccination status of the mother and time of the pregnancy.**

**During the campaign**

**
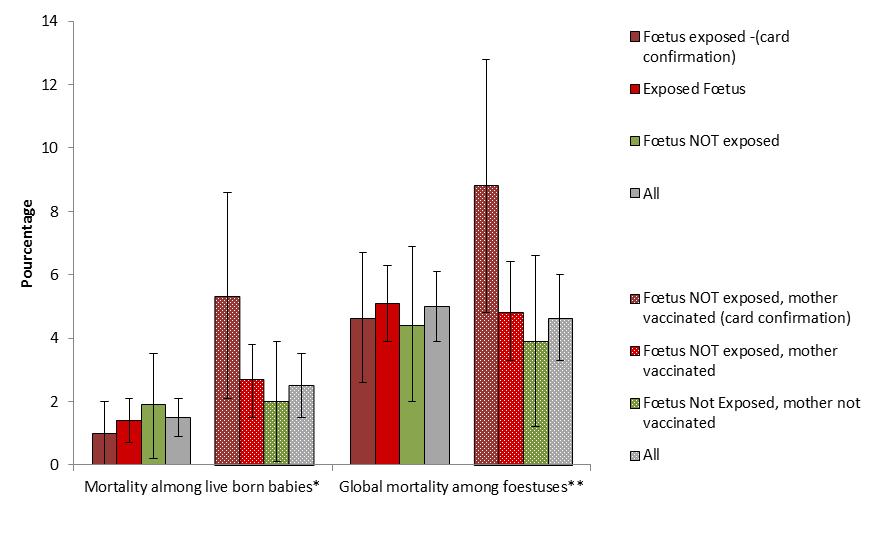
**

**After the campaign**

# S8: Structure of the cohort on 18th April 2012

Finally, we compared the distribution of the women according to their vaccination status and their month of pregnancy at the first day of the first round of the vaccination campaign (18th April 2012). If the proportion of women in their 1st trimester would have been higher for vaccinated women, then the risk of pregnancy loss associated with the vaccination could have been overestimated, and a survival model should have been usede to analyze the risk of pregnancy loss.

In our cohort, the distribution of the women according to their vaccination status and their month of pregnancy at the time of potential exposure was not significantly different (Table H).

**Table H: Distribution of the women according to their vaccination status and their month of pregnancy at the first day of the first round of the vaccination campaign (18th April 2012), according to their vaccination status.**

| **Month of pregnancy on 18^th^ April 2012** | **Unvaccinated women** | | **Vaccinated women** | |
| --- | --- | --- | --- | --- |
|  | **N** | **%** | **N** | **%** |
| 0 | 37 | 17.05% | 156 | 13.23% |
| 1 | 30 | 13.82% | 161 | 13.66% |
| 2 | 33 | 15.21% | 172 | 14.59% |
| 3 | 25 | 11.52% | 136 | 11.54% |
| 4 | 17 | 7.83% | 110 | 9.33% |
| 5 | 17 | 7.83% | 103 | 8.74% |
| 6 | 18 | 8.29% | 114 | 9.67% |
| 7 | 26 | 11.98% | 113 | 9.58% |
| 8 | 12 | 5.53% | 102 | 8.65% |
| 9 | 2 | 0.92% | 12 | 1.02% |
